# SCAPIS 2 spectrum: rationale, objectives and design of a microvascular dysfunction sub-study within the Swedish CArdioPulmonary bioImage study.

**DOI:** 10.3389/fcvm.2026.1899591

**Published:** 2026-09-24

**Authors:** Jacob Widaeus, Kerstin Welén Schef, Tomas Jernberg, Mattias Windå, Fredrik Salomonsson, Henrik Johansson, Jannike Nickander, Helge Brandberg, Maria Cronhjort, Sara Tehrani

**Affiliations:** 1 Department of Clinical Sciences, Danderyd Hospital, Karolinska Institutet, Stockholm, Sweden; 2 Department of Medical Specialties, Danderyd Hospital, Stockholm, Sweden; 3 Department of Cardiology, Danderyd Hospital, Stockholm, Sweden; 4 Neko Health AB, Stockholm, Sweden; 5 Department of Clinical Physiology, Karolinska University Hospital, Stockholm, Sweden; 6 Department of Molecular Medicine and Surgery, Karolinska Institutet, Stockholm, Sweden; 7 Department of Anaesthesia and Intensive Care, Danderyd Hospital, Stockholm, Sweden

**Keywords:** angina with no obstructive coronary arteries, cardiovascular disease, diastolic dysfunction (DD), metabolic syndrome & type II diabetes, microvascular function, obstructive coronary artery disease (OCAD)

## Abstract

The microvasculature forms the interface for nutrient exchange and signaling between the blood and tissues. Its dysfunction is increasingly recognized as a key mechanism underlying various cardiovascular diseases, including heart failure, ischemic heart disease, atherosclerosis, stroke and vascular dementia. Early detection of these subclinical processes using non-invasive assessments could facilitate timely interventions and provide mechanistic insights to prevent disease progression. SCAPIS 2 Spectrum is a prospective cross-sectional phenotyping sub study of the Swedish Cardiopulmonary Bioimage Study, recruiting up to 900 participants aged 58–75 years. The study is organized into four overlapping phenotype groups: (1) those with obstructive coronary artery disease, (2) angina with non-obstructive coronary arteries, (3) metabolic syndrome with type 2 diabetes, and (4) those with diastolic dysfunction, each compared with controls drawn from the same cohort. All participants undergo a comprehensive skin microvascular assessment at rest and following various local provocations and those without exclusion criteria or obstructive coronary artery disease will also undergo stress cardiovascular magnetic resonance imaging to assess myocardial perfusion. Descriptive statistics, association analysis, multivariable regression and exploratory modelling will be employed to investigate associations between device-derived parameters in skin microcirculation and myocardial perfusion. Circulating biomarkers will be incorporated as candidate features in exploratory analysis. Ethical approval has been obtained from the Swedish Ethical Review Authority and the Swedish Medical Products Agency (CIV-ID 23-12-045355). Study results will be disseminated through peer-reviewed publications, national and international conference presentations.

**Clinical Trial Registration:**

https://clinicaltrials.gov/study/NCT07359768?term=scapis%202%20spectrum&viewType=Card&rank=1&tab=study, ClinicalTrials.gov ID: NCT07359768.

## 1 Introduction

Microvascular dysfunction has emerged as a key mechanism in the development of cardiovascular disease (CVD). It is associated with various CVD subtypes and complications, including heart failure, ischemic heart disease, atherosclerosis, stroke and vascular dementia (1–5). Chronic low-grade inflammation, which is closely linked to metabolic syndrome, is considered a fundamental driver of systemic microvascular dysfunction and may underlie CVD. With the predicted increase in type 2 diabetes, hypertension, and obesity, inflammation-driven microvascular dysfunction is expected to rise. Early detection of microvascular dysfunction is valuable for identifying early abnormalities that precede organ damage or clinical symptoms, and it may lay the foundation for timely interventions that prevent disease progression (6–8).

The Swedish CArdioPulmonary bioImage Study (SCAPIS) is a national population-based cohort designed to improve prediction and prevention of cardiovascular and pulmonary disease. In SCAPIS 1 (2013–2018), 30,154 randomly selected adults in Sweden, aged 50–64 years underwent extensive cardiopulmonary phenotyping, including coronary computed tomography angiography (CCTA), spirometry, electrocardiography, accelerometry, questionnaires, and comprehensive biochemical profiling (9). SCAPIS 2 (2024–2026) re-examines approximately 15,000 of these individuals 8–10 years later using a largely identical protocol to assess longitudinal changes in atherosclerosis, cardiovascular aging, and lung function, and to identify risk factors for disease progression (ClinicalTrials.gov ID NCT06679777). In addition, several sub-studies further investigate participants with echocardiography, liver elastography, cerebral computed tomography, and more.

In this substudy of two novel non-invasive medical devices, we will evaluate microvascular dysfunction across a spectrum of CVD in the SCAPIS 2 cohort. We hypothesize that improved characterization of microcirculatory impairment may enhance understanding of pathophysiological mechanisms in different CVD phenotypes and provide a basis for future risk stratification and treatment monitoring. To address this, the study aims to assess the ability of these novel non-invasive methods to detect and characterize microvascular dysfunction in high-risk cardiovascular populations and to investigate associations with cardiovascular magnetic resonance imaging (CMR).

Given the complexity of microvascular pathology, our design employs a multivariable approach integrating physiological assessments with precision blood biomarkers. The overarching aim is to evaluate the investigational devices as a modality for microvascular function assessment, particularly basal microcirculatory perfusion and oxygenation, vasomotion, vasodilatation capacity and vasoreactivity through endothelial-dependent and independent pathways.

Our study objectives are to:

- Examine and compare microvascular dysfunction across predefined cardiovascular phenotypes: obstructive coronary artery disease (OCAD), angina with non-obstructive coronary arteries (ANOCA), metabolic syndrome (MetS) with type 2 diabetes and diastolic dysfunction (DD).
- Investigate associations between non-invasive microvascular parameters as measured with investigational devices and myocardial perfusion assessed by stress-CMR.
- Develop a multi-modal microvascular dysfunction risk profile integrating device-derived skin microvascular parameters with circulating plasma biomarkers.

## 2 Methods

### 2.1 Design, participants and sample sizes

SCAPIS 2 Spectrum is a single-center prospective, cross-sectional study organized into four phenotype groups and conducted as a sub-study within SCAPIS 2. Recruitment is done exclusively from the Stockholm section of SCAPIS 2, comprising an estimated 2,500 participants aged 58–75 years from Stockholm county. The SCAPIS 2 inclusion criteria and study protocol are available elsewhere (ClinicalTrials.gov ID NCT06679777).

The four study groups are defined by unique inclusion and exclusion criteria (Table 1, Figure 1). Phenotype group inclusion is conducted in parallel to minimize selection date bias, balance external influences, and distribute handling errors evenly across groups. Data used for group allocation are derived from the SCAPIS 2 main study and the SCAPIS 2 sub-study for echocardiography data. Overall exclusion criteria are extensive scarring or tattoos over the forearm, or arm amputations that preclude examination by investigational devices. Group allocation is not mutually exclusive; a participant meeting inclusion criterion for more than one disease group is included in all applicable groups in an overlapping fashion. This reflects the biological reality that these conditions co-occur (e.g., metabolic syndrome with diastolic dysfunction).

**Table 1:** Inclusion and exclusion criteria.

Cohort	n	Inclusion Criteria	Exclusion Criteria
General cohort	900^a^	Participation in SCAPIS 2 General Cohort; performed CT coronary angiography of adequate quality	Scarring, tattoos, or amputations that preclude device examination
CMR groups	385^b^	-	CAD-RADS ≥ 3, AV-block 2 or 3, left bundle-branch block or bi-fascicular block; electronic bodily implants (pacemaker, ICD, CRT); metallic foreign bodies not deemed safe, reactive airways (COPD/asthma); CKD with eGFR <30
OCAD	200	CAD-RADS ≥ 3	-
ANOCA	80	CAD-RADS < 3; angina as defined by the Rose questionnaire	Previous coronary revascularization (percutaneous coronary intervention or coronary artery bypass grafting)
MetS with diabetes type 2	180	Diabetes mellitus type 2 (14) and metabolic syndrome (15)	-
DD	125	Diastolic dysfunction as per BSE 2024 guidelines (18)	-

Description of inclusion and exclusion criteria for general cohort, phenotype groups participating in CMR investigation and all individual groups. n = number of estimated study participants. CKD, Chronic Kidney Disease; eGFR, Estimated glomerular filtration rate; CAD-RADS, Coronary artery disease reporting and data system; CT, Computed tomography; ICD, Implantable cardioverter-defibrillator; CRT, Cardiac resynchronization therapy; COPD, Chronic obstructive pulmonary disease; CMR, Cardiac magnetic resonance imaging; BSE, British Society of Echocardiography; OCAD, Obstructive coronary artery disease; ANOCA, Angina with non-obstructive coronary arteries; MetS, Metabolic syndrome; DD, Diastolic dysfunction.

a n is calculated as the maximum possible sum of participants.

b maximum possible sum of participants undergoing stress CMR.

**Figure 1:**
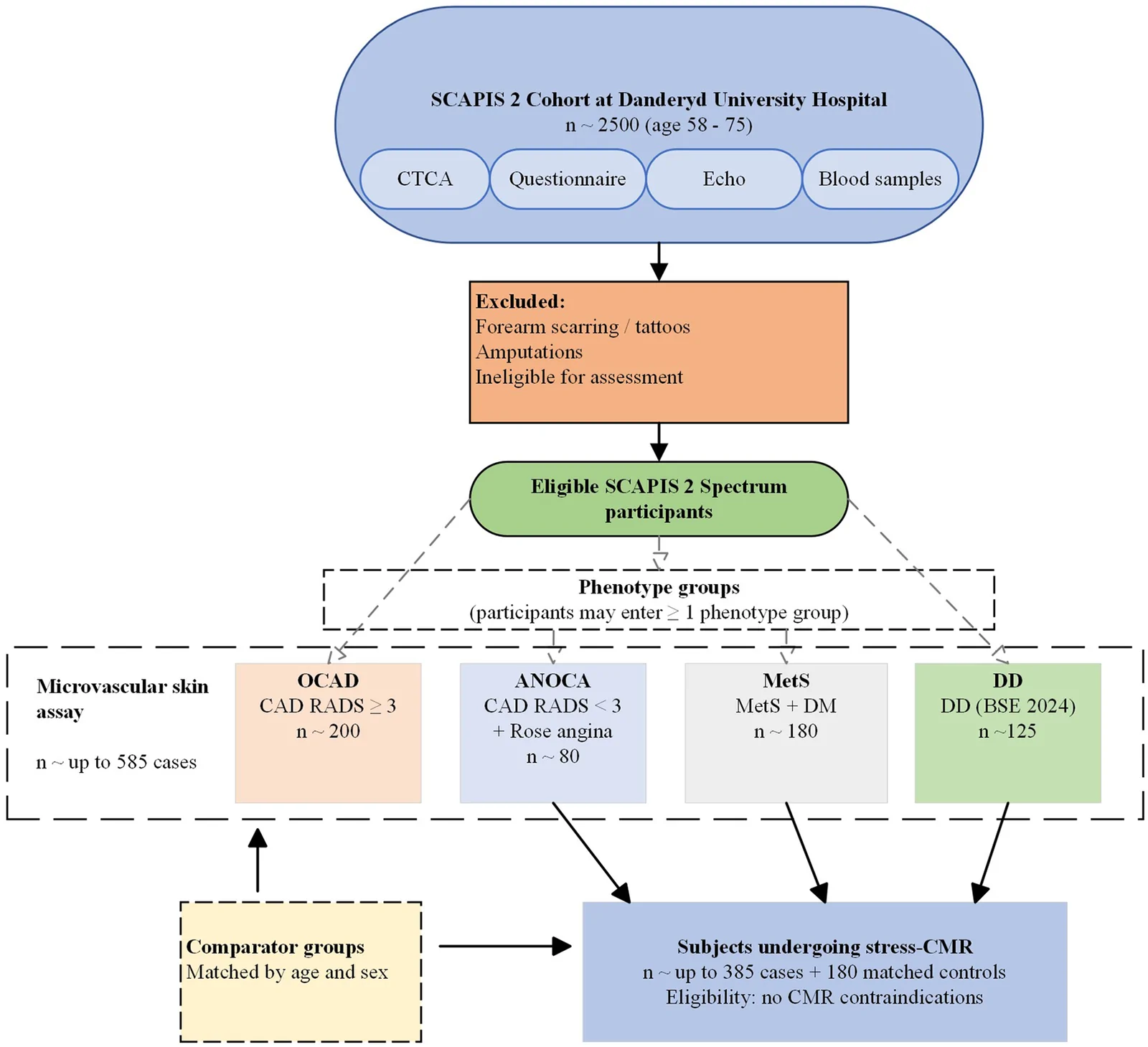
Flowchart illustrating the SCAPIS 2 spectrum study design and participant flow. Eligible participants aged 58–75 are recruited from the SCAPIS 2 cohort at Danderyd University Hospital. After excluding individuals with forearm scarring, tattoos, or amputations, participants are allocated into four phenotype groups for microvascular skin assessment: Obstructive Coronary Artery Disease (OCAD), Angina with Non-Obstructive Coronary Arteries (ANOCA), Metabolic Syndrome (MetS), and Diastolic Dysfunction (DD). Age- and sex-matched phenotype-free controls, along with a physiological reference group subset, will be utilized for comparisons. Finally, eligible participants from the ANOCA, MetS, DD groups and controls undergo stress cardiac magnetic resonance (CMR) imaging, while the OCAD phenotype group is excluded from CMR assessment.

Recruitment and data collection are planned from June 2024 to December 2026 or until all planned sample sizes have been reached. Participants are recruited exclusively from the Stockholm section of SCAPIS 2. All enrolled participants undergo the skin microvascular assessment assay, and a subset of participants are invited to undergo adenosine-stress-CMR.

Before any study-specific procedures, each potential participant receives verbal and written information about the study and is given sufficient time to consider participation. Written informed consent is obtained prior to inclusion, in accordance with EU Medical Device Regulation (MDR 2017/745) and ISO 14155:2020. Results will be reported in accordance with Strengthening the Reporting of Observational Studies in Epidemiology (STROBE) guidelines (10).

#### 2.1.1 Phenotype groups and eligibility

**Obstructive coronary artery disease.** Inclusion is based on burden of coronary atherosclerosis as measured by CCTA using the Coronary Artery Disease Reporting and Data System (CAD-RADS). Participants with CAD-RADS ≥ 3, defined as ≥50% coronary artery stenosis, are eligible for inclusion. Based on SCAPIS 1 data (11), approximately 5% of participants had CAD-RADS ≥ 3; with aging, this proportion is expected to approximately double to 10% (≈250 participants) in SCAPIS 2. The planned sample size is up to 200 participants.

**Angina with non-obstructive coronary arteries.** Inclusion requires angina as defined by the Rose Questionnaire (12) and CAD-RADS < 3. Participants with previous coronary revascularization are excluded, as percutaneous coronary intervention or bypass grafting precludes classification of the coronary anatomy as non-obstructive. In SCAPIS 1 data, the prevalence of ANOCA, using the same criteria, was 2.8% (13), yielding approximately 80 eligible participants in this substudy cohort among the estimated 2,500, after accounting for older age. The planned sample size is up to 80 participants.

**Metabolic syndrome.** Inclusion requires diagnosis of type 2 diabetes mellitus, either self-reported or diagnosed through investigations within SCAPIS 2, according to the 2025 American Diabetes Association criteria (14), and metabolic syndrome according to the 2009 American Heart Association criteria (15). Based on a 9.5% baseline prevalence of type 2 diabetes in the older Swedish population combined with a 77% rate of concomitant metabolic syndrome among these diabetic patients, the overall prevalence in this demographic is estimated at approximately 7% (16, 17). The planned sample size is up to 180 participants.

**Diastolic dysfunction.** Inclusion requires diastolic dysfunction of any grade, classified by an automated implementation of the 2024 British Society of Echocardiography (BSE) algorithm (18). In sinus rhythm, ejection fraction and global longitudinal strain determine which BSE pathway is applied. Classification within that pathway rests on three core criteria: average E/e′ (>14), left atrial volume index (>34 mL/m^2^), and peak tricuspid regurgitation velocity (>2.8 m/s); impaired relaxation is identified using age-specific e′ thresholds and left atrial reservoir strain is added where these core criteria are discordant. Participants in atrial fibrillation at the time of examination are classified by the corresponding BSE pathway. Examinations in which too few criteria are evaluable to apply the algorithm, or which the algorithm classified as indeterminate, are handled according to rules prespecified in statistical analysis plan. Based on population-based European screening studies, the prevalence of diastolic dysfunction in individuals aged 58-75 years is estimated at approximately 5%–15%, depending on the echocardiographic criteria applied (19–21). The planned sample size is up to 125 participants.

Group sizes are determined by estimated phenotype prevalence within the SCAPIS 2 Stockholm cohort and by recruitment feasibility. With planned sample sizes and 1:1 matching of controls, a standardized between-group difference is estimated with approximate 95% confidence interval half-width of ±0.20 standard deviation (SD) for OCAD, ±0.21 SD for MetS with type 2 diabetes, ±0.25 SD for DD and ±0.31 SD for ANOCA (22–25). Acetylcholine-mediated skin microvascular responses in prior studies of comparable populations differ by d ≈ 0.79–1.5; these derive from clinical cohorts compared against controls and will attenuate against phenotype-free comparators in a screening cohort, but the available sample is estimated to remain adequate for moderately attenuated effects below the reported magnitudes.

#### 2.1.2 Controls

Two nested comparator populations will be defined in the selection and presentation of data. Both are drawn from SCAPIS 2 Stockholm participants who meet none of the inclusion criteria for the four phenotype groups.

The phenotype-free comparator pool comprises participants not defined by phenotype groups. This pool is therefore a residual population from which OCAD, ANOCA, MetS with type 2 diabetes and DD are removed. Phenotype-free controls are age- and sex-matched to each phenotype group using the MatchIt package in R (26), with exact matching on sex and nearest pair matching on age (caliper 0.2 SD, rounded to the nearest year), without replacement.

The physiological reference group is a subset of the phenotype-free comparators, further selected to represent intact cardiometabolic physiology and to provide a reference distribution for device-derived measures and serum biomarkers. To ensure this group reflects the intended physiology, participants are excluded for any of the following cardiovascular criteria: heart failure, previous myocardial infarction, stroke, peripheral arterial revascularization, any previous cardiac surgery or percutaneous coronary intervention, self-reported valvular heart disease, and hypertension. Hypertension is defined by self-report, antihypertensive medication use, or mean office blood pressure ≥ 140/90 mmHg, taken as the mean of the second and third of three seated readings after 5 min of rest. Metabolic exclusions are diabetes, HbA1c ≥ 42 mmol/mol, body mass index ≥ 30 kg/m^2^, and lipid-lowering medication. Renal exclusion is defined as an estimated glomerular filtration rate < 60 mL/min/1.73 m^2^. Current smokers are also excluded from this group.

The two comparator groups support different forms of analysis. Comparisons against the phenotype-free pool estimate the increment associated with a phenotype relative to participants free of all four phenotypes. In contrast, comparisons against the physiological reference group characterize the distribution and dynamic range of the measured parameters.

The composition and adequacy of both control pools will be re-evaluated at regular intervals, including stratification for stress-CMR eligibility. Phenotype-free controls will be matched to cases at a target ratio of 1:1 where feasible within the eligible SCAPIS 2 Stockholm sub-cohort. As the matched-control pool is bound by the available phenotype-free participants in this finite cohort, the achieved ratio may vary by group; the achieved ratio per group and its implications for analytical precision will be reported.

### 2.2 Non-invasive microvascular assessment assay

Skin microvascular function is assessed using three complementary non-invasive optical modalities at rest and during standardized provocations. Variables span resting perfusion and oxygenation, provocation-induced reactivity, recovery dynamics, and frequency-domain features of the microvascular signal as well as wavelet-based signal decomposition as described by Stefanovska et al. (27).

#### 2.2.1 Investigational and comparator devices

The Perimed PeriFlux 6000 Enhanced Perfusion and Oxygen Saturation (EPOS; Perimed AB, Järfälla, Stockholm, Sweden) is used as a single-point method for a high-frequency multi-modal assessment of the dermal microcirculation employing diffuse reflectance spectroscopy (DRS) and laser Doppler flowmetry (LDF) with a sampling frequency of 20 Hz. Signal spectra yield hemoglobin oxygen saturation (%), red blood cell tissue fraction (%), and perfusion (%RBC×mm/s), the latter resolved into three speed components (0-1, 1-10, >10 mm/s) using a model based on artificial neural networks (28). It will be evaluated in conjunction with heat provocation and post-occlusive reactive hyperemia, in addition to resting values. This device is CE-marked as a research device, is used within its intended purpose and will be used as a comparator.

The PeriCam MultiFlow (Perimed AB, Järfälla, Stockholm, Sweden) performs high-frequency imaging of the dermal perfusion, using multi exposure laser speckle contrast imaging (MELSCI), combined with assessment of the blood oxygen saturation, using multi spectral imaging (MSI). It is a next-generation instrument based on the earlier laser speckle contrast imaging instruments. MELSCI yields speed-resolved perfusion in absolute units of % RBC×mm/s, estimated by an artificial neural network trained on Monte Carlo-simulated multi-exposure contrast data, in speed intervals of 0-1, 1-10 and >10 mm/s (29), with a sampling frequency of 16 Hz. It will be evaluated in conjunction with iontophoresis with acetylcholine (ACh) and sodium nitroprusside (SNP), as well as post-occlusive reactive hyperemia, in addition to resting values. This device is currently under evaluation as a class IIa non-invasive medical device within this study and is used in accordance with EU MDR and ISO 14155:2020 requirements.

Tissue Composition Imaging (TCI; Neko Health AB, Danderyd, Stockholm, Sweden) employs spatial frequency domain imaging technology (SFDI) for chromophore concentration measurements in the skin at a sampling rate of roughly 0.5 Hz. It yields chromophore concentrations of oxy-, deoxy-, and total hemoglobin and derived oxygen saturation at two sampling depths with water and melanin at discrete depths. It will be evaluated in conjunction with heat provocation and post-occlusive reactive hyperemia, in addition to resting values. This device is currently under evaluation as a class IIa non-invasive medical device within this study and is used in accordance with EU MDR and ISO 14155:2020 requirements.

#### 2.2.2 Iontophoresis

In order to distinguish between endothelial-dependent and endothelial-independent vasodilatation pathways, ACh and SNP will be administered locally and non-invasively on the skin through iontophoresis. The skin will be carefully cleaned using one swipe of a single-use isopropyl alcohol swab (70%). Two iontophoresis chambers (1.5 cm^2^ LI 611 Drug Delivery electrode, Perimed AB, Järfälla, Stockholm, Sweden) are placed in the middle portion of the volar side of the forearm, avoiding hair and overt blood vessels, spaced 5 centimeters apart. Final concentrations of 2% ACh (Sigma-Aldrich, Saint Louis, USA) and 2% SNP (Nipruss, Oresund Pharma, Copenhagen, Denmark) diluted in sodium chloride solutions are injected in chambers (0.4 mL). PeriIont device for iontophoresis (Perimed AB, Järfälla, Stockholm, Sweden) is used, connecting color-coded anode connection to proximal chamber filled with ACh and cathode to distal chamber filled with SNP, applying 0.02 mA during 200 s equivalent to 0.013 mA/cm^2^ or 2.67 mC/cm^2^ without current ramping. Iontophoresis will not be performed on participants with electronic bodily implants, in accordance with manufacturer instructions. Drug concentrations and exposure duration were selected to deliver a charge sufficient to reach plateau endothelium-dependent and endothelium-independent responses while keeping charge density below the threshold above which non-specific, current-induced vasodilatation becomes appreciable (30, 31).

#### 2.2.3 Post-occlusive reactive hyperemia

An occlusion cuff is placed on the participant's upper arm and inflated to a fixed supra-systolic pressure of 240 mmHg using digital pressure gauge guided filling, within an automated sequence. A fixed pressure with a margin to assumed distribution of blood pressure is used as the hyperemic response is unaffected by cuff pressure across 160–220 mmHg provided the applied pressure exceeds systolic blood pressure (32). After 5 min of occlusion the cuff is rapidly deflated and the ensuing hyperemia recorded. The provocation analysis will be omitted in participants whose systolic pressure at the study visit exceeds the applied cuff pressure, as complete arterial occlusion cannot then be assumed.

#### 2.2.4 Heat provocation

Pre-heated probes are placed on skin using thermally conductive tape for 10 min in order to provoke maximum heat-related hyperemia. Two probes, one with 35 and one with 42 degrees Celsius, are placed on the volar forearm. For EPOS, measurements can take place during heat provocation, at 35 and subsequently at 42 degrees Celsius for 10 min respectively.

#### 2.2.5 Protocol

Participants are asked to refrain from non-steroidal anti-inflammatory drugs for 5 days, nicotine products, exercise for 4 h and tea or caffeine for 2 h before investigation. Study participants will be resting for at least 15 min before investigation. The investigation will take place with the participants in the supine position in a room with a temperature of 21 to 23 degrees Celsius and dim lighting. Investigations are conducted in a standardized fashion by trained biomedical scientists with on-screen prompting instructions from a graphical user interface.

Left arm: MultiFlow device is placed at 22 ± 2 centimeters distance using laser-guided distance measurement. Two minutes of resting flow is recorded with subsequent iontophoresis provocation. Perfusion data is recorded during 11 min after iontophoresis start. Subsequent 5-minute arterial occlusion starts automatically and the hyperemia response is recorded for 5 min. Further, a 10-minute resting recording is performed on volar side of forearm and palm.

Right arm: TCI device is placed at 40 ± 1 centimeters distance using laser-guided distance measurement, covering the volar side of the forearm and part of the palm. The EPOS device is placed on the volar side of the right forearm ∼10 centimeters distal of the antecubital fossa, outside the field of view of the TCI device. Ten minutes of resting state is recorded with TCI and EPOS, followed by heat provocation and 10 min of recording post-heating. Subsequent 5-minute arterial occlusion starts automatically and the hyperemia response is recorded for 5 min on both TCI and EPOS devices.

The physiological constructs, signal domains and endpoint classes derived from each provocation window are summarized in Table 2; the corresponding endpoint definition, calculation and justification for each modality and domain will be specified in the subsequent statistical analysis plan. Post-occlusive reactive hyperemia elicits a global provocation response in the tissue, in contrast to the localized effects of iontophoresis and local thermal hyperemia. In healthy participants, previous studies with similar provocation protocols report hyperemic flare areas ranging from 3.4 to 5.5 cm^2^ for local heating and 0.9 to 2.5 cm^2^ for iontophoresis (33, 34). Consequently, preceding local provocations are deemed not to affect the post-occlusive response, provided the regions of interest are placed at a sufficient distance, at least 2 centimeters, from those local sites.

**Table 2:** Endpoint categories of the non-invasive microvascular assay.

Measurement window	Physiological construct	Signal domains	Endpoints categories
Resting recording	Basal perfusion, oxygenation and flowmotion	Perfusion, oxygen saturation and chromophore concentration	Average values, distribution across speed-compartments, spatial heterogeneity, frequency domain band power, relative band contribution
Acetylcholine iontophoresis	Endothelium-dependent vasodilatation	Perfusion and oxygen saturation	Pre-provocation baseline, peak response, time to peak, magnitude of change and area under the curve over defined time frame
Sodium nitroprusside iontophoresis	Endothelium-independent vasodilatation	Perfusion and oxygen saturation	As for acetylcholine
Local thermal hyperemia (35 °C, 42 °C)	Composite thermal hyperemia comprising axon-reflex and nitric oxide-dependent components; vasodilatory capacity	Perfusion, oxygen saturation and chromophore concentration	Peak reactivity, time to peak, chromophore concentration alterations during heat provocation and reactivity dynamics, including shift between velocity ranges and frequency bands in flowmotion analysis.
Arterial occlusion, ischemic phase	Tissue oxygen extraction under ischemia	Oxygen saturation	Rate of oxygen desaturation
Post-occlusive reactive hyperemia	Reactive hyperemic capacity and recovery dynamics	Perfusion, oxygen saturation and chromophore concentration	Peak response, time to peak response, area under the curve over defined time frame and rate of recovery and reactivity dynamics, including shift between velocity ranges and frequency bands in flowmotion analysis.

Description of endpoint categories derived by non-invasive microvascular assay within the scope of SCAPIS 2 Spectrum, categorized according to physiological constructs, signal domains and endpoint categories. Specific endpoints, analysis windows, normalization and the endpoint hierarchy are defined in the analysis-specific statistical analysis plans. Endpoints may be expressed in absolute units, as change from or ratio to pre-provocation baseline or biological zero, as cutaneous vascular conductance, or relative to the 42 °C response. Frequency-domain endpoints are bounded by instrument sampling frequency and recording duration, and spatial endpoints are available from imaging modalities only.

The provocations used in conjunction with the non-invasive microvascular assay may produce adverse events. Anticipated adverse events include but are not limited to: sensations of discomfort and redness of the skin for iontophoresis, discomfort during cuff-occlusion and dermal irritation as a result of heating provocation of the skin. These are anticipated to be mild, reversible and of short duration. In accordance with ISO 14155:2020, the participant may at any time cease the investigation and all adverse events or adverse device effects are collected, and when appropriate, reported to appropriate authorities.

#### 2.2.6 Data collection

Relevant data-points related to medications, co-morbidities and demographics will be collected from the SCAPIS 2 main study. In addition, data regarding Fitzpatrick scale for skin type, room temperature, skin temperature, blood pressure and changes in medications are collected during the microvascular skin assessment visit.

#### 2.2.7 Reliability testing

To evaluate the test-retest reliability of microvascular measures we will conduct a methodological validation study based on the statistical framework established by Walter et al. (35). A total of 40 healthy participants from the physiological reference group will be recruited and assessed under standardized conditions across two separate test days within 7 days. The validation will include microvascular measures derived from the study protocol. This sample size provides 80% statistical power (with *α* = 0.05) to reject a minimally acceptable reliability threshold (null hypothesis) of p_0_ = 0.40, provided the true intraclass correlation coefficient (ICC) of the method is p_1_ ≥ 0.70. Utilizing p_1_ ≥ 0.70 as the expected reliability is a conservative and defensible assumption; previous validation studies utilizing conventional single-exposure laser speckle contrast imaging with standardized single-pulse ACh iontophoresis have demonstrated excellent inter-day reproducibility, yielding peak ICC values of 0.87 (36).

Relative reliability will be assessed using ICCs. To complement these estimates, we will also quantify absolute reliability using within-subject coefficient of variation, standard error of measurement and minimal detectable change.

### 2.3 Serum biomarkers

A panel of circulating serum biomarkers across complementary platforms will be analyzed. Biomarkers include nuclear magnetic resonance-based quantification of lipid and metabolic markers, multiplex immunoassay for inflammatory cytokines, endothelial and glycocalyx markers, vascular adhesion molecules, oxidative stress markers, and hemostatic and coagulation factors, complemented by markers of pericyte and neuronal injury, and by established cardiac markers including high-sensitivity troponin T and NT-proBNP. Cellular microparticle profiling by flow cytometry additionally characterizes circulating endothelial, pericyte, and immune cell subpopulations.

#### 2.3.1 Venipuncture and sample handling

After a 15-minute rest period, venipuncture is performed, and blood is drawn into sodium citrate Vacutainer tubes, a serum-separator tube, and EDTA Vacutainer tubes. Immediately after venipuncture, all tubes are gently inverted 5–10 times. Two of the sodium-citrate tubes and all four EDTA tubes are centrifuged at 2000g for 20 min at 20 °C, followed by plasma extraction and storage at −80 °C. The remaining two sodium-citrate tubes are first centrifuged at 2,500 g for 15 min at 20 °C, plasma is extracted, and the plasma is then re-centrifuged at 2,500 g for 15 min at 20 °C for platelet-free plasma, before final storage at −80 °C. The serum-separator tube is allowed to clot for 30 min, and is then centrifuged at 2000g for 10 min at 21 °C, followed by serum aliquoting and storage at −80 °C.

### 2.4 Stress cardiovascular magnetic resonance imaging

#### 2.4.1 Inclusion and exclusion criteria

All participants enrolled in the study may be eligible for CMR. Participants abstain from caffeine for 24 h and nicotine for 12 h before examination, with adherence control via verbal confirmation before investigation. Due to difficulties interpreting quantitative perfusion of myocardial perfusion reserve (MPR) in CMR imaging and safety concerns, the following exclusion criteria are implemented:

i) Obstructive coronary arteries as defined by CAD-RADS ≥ 3 as this may produce perfusion deficits not related to endpoints.
ii) Atrioventricular conduction disorders that preclude intravenous adenosine administration.
iii) Left bundle branch block or bi-fascicular block.
iv) Chronic obstructive pulmonary disease and severe asthma that preclude intravenous adenosine administration.
v) Participants with foreign bodies that may constitute a risk with investigation in accordance with MR safety screening procedures, such as intraocular shrapnel.
vi) Electronic bodily implants such as cardiac pacemakers or deep brain stimulators.
vii) Estimated glomerular filtration rate below 30 mL/min/1.73 m^2^ that precludes gadolinium enhancement.
viii) Participants using dipyridamole, theophylline or other agents interfering with adenosine-vasodilatation.

#### 2.4.2 Acquisition and perfusion analysis

Eligible participants from the study groups will undergo stress-CMR-perfusion at 1.5 T at Karolinska University Hospital, performed by experienced personnel. The study protocol includes quantitative first-pass perfusion imaging during adenosine stress and at rest (following an at least 10-minute pause after adenosine discontinuation), for assessment of the myocardial perfusion. This perfusion imaging will utilize the optimized dual-sequence method described by Kellman et al. (37), employing a fast low-angle shot readout for the arterial input function and a balanced steady-state free precession readout optimized for the myocardium. Three short-axis slices (basal, mid and apical) will be acquired. Adenosine (Adenosin®, Life Medical AB, Stockholm, Sweden) will be infused over 5 min at 140 µg/kg/min; if there is an inadequate hemodynamic response (defined as a heart rate increase of <10 beats per minute), the dose will be gradually increased to 210 µg/kg/min. For first-pass imaging, an intravenous bolus of contrast agent will be administered (gadobutrol at 0.05 mmol/kg, with a total contrast dose of 0.15 mmol/kg utilized for the complete study). Separate venous cannulas will be used for the adenosine infusion and the contrast agent.

Cine imaging covering the entire left ventricle in short-axis slices and three long-axis views provide left ventricular volumes and mass reflecting cardiac dimensions and pump function. Native T1, native T2, and extracellular volume mapping, alongside late gadolinium enhancement imaging acquired 10-25 min following contrast administration, will provide additional tissue characterization. Flow quantification of the pulmonary artery and aorta allows for precise quantification of stroke volumes.

Image analysis will be conducted on a dedicated post-processing workstation by a reader blinded to all patient information. Perfusion maps will be created by an inline, fully-automated software using the online Gadgetron reconstruction framework (38), calculating myocardial perfusion values (mL/min/g) based on the blood-tissue exchange model, established by Bassingthwaighte et al. (39). Values quantified by inline artificial intelligence will be reported globally and regionally according to the 16-segment model. Participants exhibiting regional perfusion defects will be excluded from global perfusion analysis as this precludes interpretation of global stress MBF and MPR as measures of microvascular function. Endocardial and epicardial contours will be drawn in all short-axis slices of the left ventricle in end-diastole to obtain left and right ventricular volumes and mass. Late gadolinium enhancement images will be used to identify areas with focal scar. Regions of interest will be drawn in native T1, native T2 and extracellular volume maps in the midventricular septum to acquire advanced tissue characteristics.

There have been several proposed cutoffs of stress perfusion and MPR around 2.2 for both (40, 41) which are similar to acquired thresholds at our institution (42, 43). However, given the lack of perfusion values in healthy people in this age-range at our institution the pre-defined cutoff for reduced stress MBF and reduced MPR as a categorical measure will be 2 standard deviations below the mean of the subjects included in the physiological reference group. Resting MBF will be reported alongside stress MBF and MPR, so that a reduced MPR driven predominantly by elevated resting perfusion can be distinguished from one reflecting an attenuated hyperemic response.

### 2.5 Statistical analysis and endpoints

Statistical analyses will be conducted according to prespecified statistical analysis plans (SAPs) finalized and published before access to the locked analysis database and before initiation of the formal analysis phase. These SAPs will define analysis populations, endpoint hierarchies, device-derived variable definitions, covariate adjustment, handling of missing data and outliers, sensitivity analyses, and procedures for multiplicity control. Peak total perfusion after ACh iontophoresis as measured by MELSCI, and MPR will be used as principal outcomes and phenotype-free controls will act as primary comparators, however each mechanistic domain depending on hypothesis will provide its own endpoint hierarchies as specified in relevant SAP. Endpoint calculations and rationale for each device derived metric will be declared and justified in relevant SAP. Sensitivity analyses for inferential comparisons will include adjustment for time of day, room and skin temperature, and Fitzpatrick skin type. Intervals between CCTA, echocardiography, the microvascular assessment visit and stress-CMR are recorded for every participant and reported as distributions rather than constrained by a fixed maximum, with interval-adjusted and interval-restricted sensitivity analyses prespecified in the relevant statistical analysis plan. Sensitivity analyses restricted to participants meeting criteria for a single phenotype group, and models including co-occurring phenotypes simultaneously, will be used to assess whether observed associations are attributable to the index phenotype rather than to multimorbidity. Models adjusting for diastolic dysfunction status are fitted in echo-verified participants, with the analyzable sample size reported per model. Further sensitivity analysis will be prespecified in the relevant SAP.

Skin microvascular parameters will be compared between each phenotype group and its matched comparators. Because phenotype groups overlap and are matched separately from a shared comparator pool, a single pairwise comparison reflects the phenotype of interest together with any co-occurring phenotypes, and comparisons across groups share participants and are therefore not independent. Each SAP will state how this overlap is handled in estimation and inference, and how narrowly the resulting estimate can be interpreted.

Associations between skin microvascular parameters and stress-CMR-derived myocardial perfusion will be assessed using correlation analyses and multivariable regression models. The number of participants invited, imaged and analyzable will be reported per phenotype group together with reasons for non-participation, and baseline characteristics will be compared between imaged participants, eligible non-participants and those excluded for contraindications. Exploratory modelling will evaluate whether device-derived skin microvascular parameters and circulating biomarkers can be integrated into mechanistically informed pathway-specific scores of microvascular dysfunction, and whether these scores are associated with stress-CMR-derived MPR in CMR-eligible participants. Resting MBF, stress MBF and MPR will primarily be analyzed as continuous measures; the categorical definitions of reduced stress MBF and reduced MPR are exploratory endpoints. Participants in ANOCA group whose CMR-derived perfusion falls below the prespecified threshold, 2 standard deviations below the mean of the physiological reference group, are designated as having impaired myocardial perfusion and are analyzed as a prespecified exploratory subgroup. Resampling for internal validation will be performed at the level of the unique participant. Reliability analyses will include measures of relative and absolute reliability, including ICCs, within-subject coefficient of variation, standard error of measurement, and minimal detectable change. Primary, secondary, sensitivity, exploratory, and *post hoc* analyses will be clearly distinguished. Multiplicity will be addressed through the prespecified analysis hierarchy and, where appropriate, formal adjustment procedures such as false discovery rate control. Effect estimates will be reported with 95% confidence intervals and exact *p*-values where relevant.

## 3 Discussion

The phenotyping of cardiovascular disease is central, as microvascular dysfunction is increasingly recognized not just as a localized issue, but as a systemic disease. We specifically selected cohorts with OCAD, ANOCA, MetS with type 2 diabetes, and DD because they represent a broad spectrum of risk profiles where microvascular impairment plays a crucial, yet diverse, pathophysiological role. For instance, chronic low-grade inflammation is considered a fundamental driver of systemic microvascular dysfunction that may underlie these varying CVD phenotypes and the cardiometabolic syndrome. By characterizing these distinct groups, we will gain knowledge beyond generalized assumptions and much-needed mechanistic insights into how systemic microcirculatory impairment is associated with these specific disease states.

To translate these mechanistic insights into routine clinical practice, there is a pressing need for accessible, non-invasive methods to investigate and risk-stratify patients. By evaluating these novel non-invasive devices, this study aims to investigate their respective contribution to the understanding of microvascular function. Furthermore, integrating these device-derived physiological assessments with precision blood biomarkers enables the exploration of a multi-modal microvascular dysfunction risk profile. Ultimately, this comprehensive, multi-organ profiling may facilitate a shift toward precision medicine.

This study has several strengths. It leverages the well-characterized SCAPIS 2 cohort with comprehensive phenotypic data, enabling disease stratification. The use of complementary non-invasive imaging modalities allows multidimensional assessment of microvascular function. Integration of quantitative stress-CMR with skin measurements provides unique examination of whether skin-based assessments reflect indices of myocardial perfusion.

Several limitations should be considered. First, the cross-sectional design supports characterization of associations rather than causal inference. Second, the fixed assignment of instruments to specific limbs may confound device-specific differences with physiological side-to-side variation and fixed provocation sequence may introduce carry-over effects. Third, the protocol generates a large number of device-derived indices; analyses are accordingly exploratory, with endpoint hierarchies and multiplicity control prespecified in the statistical analysis plans, and individual associations are hypothesis-generating until replicated. Fourth, because the phenotype groups overlap and share comparators, pairwise contrasts will be interpreted with co-occurring conditions and dependence between contrasts in mind. Fifth, stress-CMR is limited to participants without contraindications or obstructive coronary artery disease and those with normal myocardial perfusion may have epicardial vasospastic disease or have non-cardiac symptoms. Moreover, stress CMR predominantly assesses endothelium-independent vasodilator function, which should be considered when relating MPR to skin microvascular indices that probe different vasomotor pathways. In those with impaired myocardial perfusion, the impairment may or may not be due to microvascular vasospasm since adenosine stress-CMR cannot exclude epicardial or microvascular spasm. Sixth, the reliability substudy is conducted in healthy volunteers, and reproducibility may differ with age, diabetes, vascular disease, or vasoactive medication. Provocation protocols and derived indices remain incompletely standardized and lack normative data. Finally, exclusion of participants with forearm scarring, tattoos, amputations, or CMR contraindications precludes generalization to these groups, and single-center recruitment from a Swedish population-based cohort further limits generalizability, particularly across Fitzpatrick skin types.

SCAPIS 2 Spectrum is designed to systematically compare skin microvascular function across a broad range of the cardiovascular disease spectrum, from atherosclerotic coronary disease to angina without obstructive coronary arteries, metabolic syndrome and diastolic dysfunction, within a single population-based cohort and against CMR-measured myocardial perfusion. By combining mechanistically targeted provocations with multimodal optical imaging and stress-CMR, it provides the infrastructure to determine whether non-invasive skin assessment can detect, differentiate and stratify microvascular dysfunction across clinically relevant CVD phenotypes. If successful, the study will provide mechanistic insights into multiple, clinically relevant, phenotypes of cardiovascular disease and may subsequently advance a precision medicine framework incorporating microvascular function.

## Dissemination

Dissemination of study results will include publication in peer-reviewed journals, in addition to presentations at national and international conferences. Decisions regarding publication, manuscript content, interpretation of findings and the final choice of statistical analyses will be made independently of instrument stakeholders, although technical input may be sought when required for device-specific data processing or interpretation of measurement outputs.
